# Body composition and behaviour in adult rats are influenced by maternal diet, maternal age and high-fat feeding

**DOI:** 10.1017/jns.2014.64

**Published:** 2015-02-04

**Authors:** S. Ware, J.-P. Voigt, S. C. Langley-Evans

**Affiliations:** 1 School of Biosciences, University of Nottingham, Sutton Bonington, Loughborough, UK; 2 School of Veterinary Medicine and Science, University of Nottingham, Sutton Bonington, Loughborough, UK

**Keywords:** Fetal programming, Obesity, Ageing, CON, maternal control diet, 18 % casein, LP, maternal low-protein diet, 9 % casein

## Abstract

Fetal exposure to maternal undernutrition has lifelong consequences for physiological and metabolic function. Maternal low-protein diet is associated with an age-related phenotype in rats, characterised by a period of resistance to development of obesity in early adulthood, giving way to an obesity-prone, insulin-resistant state in later adulthood. Offspring of rats fed a control (18 % casein) or low-protein (9 % casein; LP) diet in pregnancy were challenged with a high-fat diet at 9 months of age. To assess whether other maternal factors modulated the programming effects of nutrition, offspring were studied from young (2–4 months old) and older (6–9 months old) mothers. Weight gain with a high-fat diet was attenuated in male offspring of older mothers fed LP (interaction of maternal age and diet; *P* = 0·011) and adipose tissue deposition was lower with LP feeding in both males and females (*P* < 0·05). Although the resistance to weight gain and adiposity was partially explained by lower energy intake in offspring of LP mothers (*P* < 0·001 males only), it was apparent that energy expenditure must be influenced by maternal diet and age. Assessment of locomotor activity indicated that energy expenditure associated with physical activity was unlikely to explain resistance to weight gain, but showed that offspring of older mothers were more anxious than those of younger mothers, with more rearing observed in a novel environment and on the elevated plus-maze. The data showed that in addition to maternal undernutrition, greater maternal age may influence development and long-term body composition in the rat.

A broad body of epidemiological evidence suggesting that the nutritional environment experienced in fetal life may programme risk of chronic degenerative diseases associated with ageing is complemented by animal studies showing prenatal programming of adult disease by maternal nutritional status^(^^1^^,^^2^^)^. In the rat, fetal exposure to a maternal low-protein diet is associated with a number of physiological outcomes that manifest in early adulthood, including raised blood pressure^(^^3^^,^^4^^)^, endothelial dysfunction^(^^5^^)^, glucose intolerance^(^^6^^,^^7^^)^ and renal impairment^(^^8^^)^.

Earlier work in our laboratory showed that some aspects of the programmed phenotype associated with maternal undernutrition develop only with ageing. Offspring of rats fed a low-protein diet in pregnancy are obesity resistant and have greater insulin sensitivity in early adulthood^(^^9^^,^^10^^)^; however, between 9 and 18 months of age they become more prone to fat gain, develop hepatic steatosis and are increasingly insulin resistant^(^^9^^)^. These observations have all been made with adult animals being fed a low-fat standard chow diet, which is specifically formulated to limit fat gain in laboratory rodents. Few studies have considered the impact of feeding a high-fat diet against a background of exposure to maternal undernutrition, and those which have did not consider the effect of high-fat feeding in older animals^(^^11^^,^^12^^)^. There is, therefore, a gap in the literature in terms of understanding how the early-life experience has an impact upon the ageing response to dietary challenge.

Previous studies of younger adult animals exposed to maternal undernutrition *in utero* have evaluated differences in metabolic phenotype and in particular body composition following maternal protein restriction. Whilst body composition has been shown to be programmed by maternal nutrition, there is no clear association between this and the programming of food intake. This has prompted interest in energy expenditure associated with physical activity and behavioural studies that have examined locomotor activity. These have produced variable outcomes that are dependent upon the precise nature of the maternal insult^(^^13^^,^^14^^)^.

One of the main advantages of working with animal models to investigate early-life programming is the capacity to control for confounding factors that are present in epidemiological studies. However, most of the extensive literature on programming in rodents has focused on a relatively narrow and focused range of maternal characteristics (standardised age, weight, housing conditions) to ensure that the effects of maternal diet are clearly identifiable. There has been very little investigation of the effects of maternal age upon the long-term health and physiology of offspring. This is an important issue as in the human population age at first childbearing is steadily increasing, with many women choosing to delay pregnancy^(^^15^^)^. This carries risk in terms of pregnancy outcome^(^^16^^,^^17^^)^, but the potential impact upon fetal development is largely uncharacterised. In rats greater age at mating is associated with smaller litter size and greater body weight in offspring at the age of weaning^(^^18^^)^, but nothing is known about the longer-term effects of greater maternal age.

The potential contribution of maternal age as a modifier of the programming effects of nutrition has been studied in sheep, but only in the context of offspring from younger mothers. Lambs born to immature ewes and exposed to maternal under- or overnutrition developed greater adiposity when allowed to overfeed^(^^19^^,^^20^^)^. This work demonstrated that maternal age has the potential to make an impact upon fetal development and contribute to early-life programming of later disease. In the present study we sought to examine two hypotheses using a well-established model of nutritional programming. We hypothesised that feeding a high-fat diet to older animals exposed to protein restriction *in utero* would advance the development of age-related metabolic disturbance, manifesting as increased adiposity. Superimposing greater maternal age onto the nutritional insult was hypothesised to exacerbate the programming effect of protein restriction.

## Materials and methods

### Animals and experimental protocol

Animal experiments were carried out under license from the UK Home Office, in accordance with the Animals (Scientific Procedures) Act 1986. Animals were housed in Perspex cages with *ad libitum* access to food and water. The animal unit operated on a 12 h light–12 h dark cycle at a temperature of 20 ± 2°C. Virgin female Wistar rats were mated at four different ages: 2 months, 4 months, 6 months and 9 months. Upon confirmation of pregnancy by the presence of a semen plug the female rats were randomly allocated to be fed either a control diet (18 % casein; CON) or a maternal low-protein diet (9 % casein; LP). These were of the same composition as those used in earlier studies in our laboratory, comprising casein-based diets, with carbohydrate provided as a 2:1 mix (w/w) of starch and sucrose. The full composition of the CON and LP diets is published elsewhere^(^^21^^)^. Weight and food intake were recorded daily for all animals during pregnancy.

Reproductive success was significantly lower in older mothers. Whereas 100 % of 2-month-old dams and 87·5 % of 4-month-old dams carried litters to full term, only 56·25 % of 6-month-old and 37·5 % of 9-month-old dams successfully littered. This reproductive success was not influenced by the maternal diet. As there were no significant differences between offspring of 2- and 4-month-old mothers, and 6- and 9-month-old mothers, for all analyses we combined 2- and 4-month groups into a ‘young’ maternal group and 6- and 9-month groups into an ‘old’ maternal group.

On giving birth, all litters were culled to a maximum of ten pups (five male, five female) and all suckling dams were fed a standard laboratory chow diet (B&K Universal). This same diet was used to wean the offspring at 4 weeks of age. The offspring were then maintained until 9 months of age when they were randomly allocated to be fed either the standard laboratory chow (14 MJ/kg digestible energy), or a high-fat diet (20 % casein, 22 % maize starch, 29·5 % butter, 10 % maize oil, 10 % sucrose, 5 % fibre, 2·5 % minerals and vitamins, 21 MJ/kg digestible energy). Where possible, one male and one female from each litter were allocated to each trial diet group and food intake and body weight were monitored weekly over a period of 9 weeks. All animals underwent behavioural testing at 10 months of age. At the end of the feeding trial the animals were killed by CO_2_ asphyxia and cervical dislocation. Adipose tissue was dissected from the perirenal and gonadal depots and carefully weighed. Samples of each depot were snap-frozen for analysis of adipocyte size.

### Adipocyte measurements

Snap-frozen samples of adipose tissue were sectioned using a cryostat and mounted onto microscope slides. Sections were photographed using Image-Pro software and the size of 300 fat cells per sample was determined following calibration against a 1 mm graticule.

### Behavioural measurements

In order to assess behaviour of the animals and whether this may make an impact upon adiposity at 10 months of age, rats were transferred from their home cage into a new, clean cage to measure locomotor activity as reported previously^(^^13^^)^. Cages sat in an array of forty-eight IR beams on two levels (Linton AMIU53 Infrared Activity Monitor) allowing monitoring of movement within the cage (breaking beams in the x–y plane) and rearing movements (breaking beams in the z plane). In the x–y plane breaking more than one beam in 1 s was defined as ‘mobility’, whilst breaking one beam within 1 s of the test was defined as ‘activity’. Mobility would include motion around the floor of the cage, whilst activity movements would include grooming, small movements and feeding. Recording of movement took place over a 90-min period, but for the purposes of the present study we report only on the first 30 min, during which the animals were habituating to a novel environment. In the subsequent 1 h rats typically went to sleep and little activity was recorded. Total beam breaks in all planes were recorded and the percentage of beam breaks indicating rearing, mobility and activity was calculated. All locomotor activity testing was conducted in a quiet room during the light phase under the standard housing conditions for the animal unit. Locomotor testing was repeated three times for each animal.

Exploratory and anxiety behaviour was examined using the elevated plus-maze test^(^^22^^)^. At 10 months of age the rats were placed on the plus-maze, which was made of black plastic and comprised two open and two closed arms (16 × 46 cm) positioned 50 cm above the floor. Closed arms had a surrounding wall height of 10 cm. The apparatus was situated in an undisturbed, isolated room and behaviour during the 5-min test was recorded by a camera positioned 100 cm above the centre of the plus-maze. EthoVision 3 software (Noldus Information Technology) was used for tracking the animals and recording the distance moved on the apparatus, arm entries and the time spent in different arms. Time spent in closed arms was interpreted as anxiety related behaviour, whilst time in the open arms represented exploratory behaviour. Elevated plus-maze testing took place between 09.00 and 13.00 hours each day (light phase), with animals placed in the testing room 30 min before testing. Light intensity in the test room was lower than normal housing conditions (130 lux).

### Statistical analysis

Data are reported as mean values with their standard errors throughout the article. Initial analysis confirmed that for all outcome measures there were significant differences between male and female offspring (*P* < 0·01) and subsequent analysis considered the sexes separately. Three-way ANOVA was performed to identify the main effects of maternal age, maternal diet and trial diet on offspring body composition, adipocyte characteristics and behaviour. Analysis of maternal data (body-weight gain, adiposity) was performed by two-way ANOVA (main effects maternal age and maternal diet). *Post hoc* testing using a Bonferroni test was performed where ANOVA indicated main effects of the fixed factors. Where interactive effects (for example, maternal age × maternal diet) were indicated *post hoc* testing was not possible. For all analyses, *P* < 0·05 was accepted as statistically significant.

## Results

At the start of pregnancy the rat dams aged 6–9 months were significantly heavier than in the 2- to 4-month-old group (Table 1), reflecting their longer pre-pregnancy growth period. The younger dams gained approximately 33 % more weight during pregnancy than the older animals. Weight gain and litter size were not influenced by the feeding of a low-protein diet, but the older dams had markedly smaller litters than the younger animals (Table 1). Despite this, the birth weights of pups born to older mothers were similar to the younger dams' litters. When the offspring were weaned, all mothers were culled for an assessment of adiposity. Older mothers had significantly larger perirenal fat stores than the younger animals, at the end of the suckling period, but parametrial fat depots were of similar size in all animals. Maternal adiposity was not affected by the maternal diet (Table 1).

**Table 1.** Maternal weight gain, adiposity and offspring birth weight§ (Mean values with their standard errors)

			Mating weight (g)§		Weight gain (g)§		Litter size (*n* pups)		Birth weight (g)		Perirenal fat (% body weight)		Perirenal fat (% body weight)	
Maternal age (months)	Maternal diet	*n*	Mean	sem	Mean	sem	Mean	sem	Mean	sem	Mean	sem	Mean	sem
2–4	CON	16	242	12	164	28	14	1	5·89	0·21	0·51	0·07	0·73	0·09
	LP	14	251	11	157	20	12	1	5·87	0·20	0·54	0·06	0·74	0·08
6–9	CON	7	335*	17	124*	21	10*	1	6·06	0·32	0·67*	0·10	0·79	0·12
	LP	7	335*	17	119*	31	8*	1	6·21	0·29	0·74*	0·10	0·95	0·12
Effect of age (*P*)			<0·001		<0·001		0·001		NS		0·04		NS	
Effect of maternal diet (*P*)			NS		NS		NS		NS		NS		NS	
Effect of maternal diet (*P*)			NS		NS		NS		NS		NS		NS	

CON, maternal control diet, 18 % casein; LP, maternal low-protein diet, 9 % casein.

* Significant effect of maternal age comparing animals of the same dietary group (*P* < 0·01).

§ Mating weight was determined on the day a seminal plug was detected and weight gain was calculated as the increase in weight from mating weight to weight on the day pups were delivered. Maternal fat pad weights were determined at the end of lactation.

Among the male offspring starting the feeding trial at 9 months of age, there was evidence of maternal diet effects upon body weight (Table 2). Generally the offspring of mothers fed the LP diet were smaller than those fed the control diet. As the animals were fully grown at the start of the study, weight gain among the males fed the chow diet was negligible over the 9-week trial. In contrast, the high-fat diet induced significant increases in weight. Weight gain of the high-fat diet-fed males born to older mothers that had been exposed to a maternal LP diet was considerably less than seen in the other high-fat-fed groups (only 56 % of weight gain seen in control offspring from older dams and 71 % of gain seen in LP-exposed offspring of younger dams; interaction of maternal age and maternal diet; *P* = 0·011). Interestingly this group was the only group of high-fat-fed males that did not consume significantly more energy than their chow-fed equivalents over the trial (Table 2), reflecting the fact that, in general, consumption of the high-fat diet was lower in LP-exposed animals than in rats exposed to the control diet *in utero*. As expected the feed efficiency (weight gained per unit energy) for most groups of males was significantly greater with high-fat feeding. The offspring of low-protein-fed older mothers did not exhibit this expected effect as there was still some weight gain in the chow-fed animals, and a lower than expected gain with the high-fat diet that was not explained in full by lower energy intake (Table 2).

**Table 2.** Weight and energy intake of male offspring during high-fat feeding trial (Mean values with their standard errors)

				Initial body weight (g)		Weight gain (g)		Total energy consumption (MJ)		Energy efficiency (g/MJ)§	
Maternal age (months)	Maternal diet	Trial diet	*n*	Mean	sem	Mean	sem	Mean	sem	Mean	sem
2–4	CON	Chow	15	721	24	10	8	29·33	3·73	0·27	0·24
		Butter	15	681	24	111‡	8	37·42‡	5·10	2·93‡	0·24
	LP	Chow	13	620†	26	5	9	25·75†	2·32	0·18	0·26
		Butter	16	645	24	92‡	8	3·25‡	3·84	2·66‡	0·23
6–9	CON	Chow	6	671	38	10	14	28·03	0·92	0·36	0·42
		Butter	6	670	38	115‡	13	40·92‡	6·85	2·93‡	0·41
	LP	Chow	7	697	35	30	13	27·71	4·34	1·17	0·78
		Butter	4	612†	47	65†‡	16	31·70†	6·00	1·99	0·47
Effect of age (*P*)				NS		NS		NS		NS	
Effect of maternal diet (*P*)				0·045		NS		<0·001		NS	
Effect of trial diet (*P*)				NS		<0·001		<0·001		<0·001	
Interaction age × maternal diet (*P*)				NS		0·011		NS		NS	
Interaction age × trial diet (*P*)				NS		NS		NS		NS	
Interaction maternal diet × trial diet (*P*)				NS		NS		NS		0·05	
Interaction age × maternal diet × trial diet (*P*)				NS		NS		0·036		NS	

CON, maternal control diet, 18 % casein; LP, maternal low-protein diet, 9 % casein.

* Significant effect of maternal age compared with animals from the same maternal diet, trial diet group (*P* < 0·05).

† Significant effect of maternal diet compared with animals from the same age, trial diet group (*P* < 0·05).

‡ Significant effect of trial diet compared with animals from the same maternal diet, and age group (*P* < 0·05).

§ Energy efficiency is defined as weight gained per MJ consumed.

In contrast to the males, the body weights, weight gain, energy efficiency and energy consumption of the female offspring were influenced only by the trial diet (Table 3). Rats fed the high-fat diet consumed significantly more energy and gained more weight than those fed the chow diet, irrespective of maternal age and maternal diet. It was apparent that, as in the males, the magnitude of the high-fat diet-induced weight gain was lower in the offspring of the LP-fed, older mothers, but this did not achieve statistical significance.

**Table 3.** Weight and energy intake of female offspring during high-fat feeding trial (Mean values with their standard errors)

				Initial body weight (g)		Weight gain (g)		Total energy consumption (MJ)		Energy efficiency (g/MJ)§	
Maternal age (months)	Maternal diet	Trial diet	*n*	Mean	sem	Mean	sem	Mean	sem	Mean	sem
2–4	CON	Chow	9	353	23	3	11	20·12	1·12	0·14	0·41
		Butter	12	361	20	101‡	10	27·62‡	0·97	3·53‡	0·26
	LP	Chow	15	351	18	3	9	19·04	0·87	0·12	0·32
		Butter	15	353	17	82‡	9	26·06‡	0·85	3·02‡	0·32
6–9	CON	Chow	5	348	28	8	15	19·53	1·50	0·41	0·55
		Butter	5	352	28	88‡	15	25·84‡	1·50	3·39‡	0·54
	LP	Chow	6	306	24	7	14	16·85	1·37	0·57	0·51
		Butter	7	337	26	44‡	13	2·67‡	1·27	1·74‡	0·47
Effect of age (*P*)				NS		NS		NS		NS	
Effect of maternal diet (*P*)				NS		NS		NS		NS	
Effect of trial diet (*P*)				NS		<0·001		<0·001		<0·001	
Interaction age × maternal diet (*P*)				NS		NS		NS		NS	
Interaction age × trial diet (*P*)				NS		NS		NS		NS	
Interaction maternal diet × trial diet (*P*)				NS		NS		NS		NS	
Interaction age × maternal diet × trial diet (*P*)				NS		NS		NS		NS	

CON, maternal control diet, 18 % casein; LP, maternal low-protein diet, 9 % casein.

† Significant effect of trial diet compared with animals from the same maternal diet, and age group (*P* < 0·05).

§ Energy efficiency is defined as weight gained per MJ consumed.

Table 4 shows the measures of adiposity in the male offspring at the end of the feeding trial. Consuming the high-fat diet resulted in significantly larger perirenal fat depots, but not gonadal fat depots. Both depots were influenced by maternal diet, with LP-exposed offspring having less fat than control offspring, regardless of maternal age. Animals born to older mothers that consumed the control diet had significantly larger perirenal adipocytes than those born to younger mothers, but this effect of maternal age was absent in the LP-exposed offspring. In contrast, LP-exposed rats born to older mothers had larger gonadal adipocytes. Adipocyte size was not influenced by the feeding of a high-fat diet.

**Table 4.** Fat pad weights and adipocyte size in male offspring (Mean values with their standard errors)

								Adipocyte diameter (μm)			
				Perirenal fat		Gonadal fat		Perirenal		Gonadal	
Maternal age (months)	Maternal diet	Trial diet	*n*	Mean	sem	Mean	sem	Mean	sem	Mean	sem
2–4	CON	Chow	15	1·50	0·07	2·04	0·16	143	10	132	7
		Butter	15	1·82‡	0·07	2·49	0·16	155	11	127	23
	LP	Chow	13	1·37†	0·08	1·53†	0·18	157	11	125	13
		Butter	16	1·76†	0·07§	2·09†	0·16	150	9	119	20
6–9	CON	Chow	6	1·60	0·12	2·06	0·28	194†	12	130	9
		Butter	6	1·67	0·12	2·38	0·27	197†	12	123	21
	LP	Chow	7	1·41†	0·11	1·97	0·26	136†	11	167*†	26
		Butter	4	1·31†‡	0·14	1·79†	0·32	149†	13	182*†	38
Effect of age (*P*)				NS		NS		0·03		0·002	
Effect of maternal diet (*P*)				0·011		0·019		0·004		0·004	
Effect of trial diet (*P*)				0·019		NS		NS		NS	
Interaction age × maternal diet (*P*)				NS		NS		<0·001		NS	
Interaction age × trial diet (*P*)				0·013		NS		NS		NS	
Interaction maternal diet × trial diet (*P*)				NS		NS		NS		NS	
Interaction age × maternal diet × trial diet (*P*)				NS		NS		NS		0·031	

CON, maternal control diet, 18 % casein; LP, maternal low-protein diet, 9 % casein.

* Significant effect of maternal age compared with animals from the same maternal diet, trial diet group (*P* < 0·05).

† Significant effect of maternal diet compared with animals from the same age, trial diet group (*P* < 0·05).

‡ Significantly effect of trial diet compared with animals from the same maternal diet, and age group (*P* < 0·05).

In contrast to the males, female adiposity was significantly influenced at both depots by the high-fat diet, with larger perirenal and gonadal fat pads and enlarged perirenal adipocytes observed regardless of maternal age and diet (Table 5). However, just as in males, the female offspring exposed to the LP diet *in utero* had less fat at these depots than those whose mothers were fed the control diet. In the gonadal fat depot an effect of the high-fat diet was noted only in the offspring of older mothers that consumed the LP diet (maternal diet × maternal age × trial diet interaction; *P* = 0·031).

**Table 5.** Fat pad weights and adipocyte size in female offspring (Mean values with their standard errors)

								Adipocyte diameter (μm)			
				Perirenal fat		Gonadal fat		Perirenal		Gonadal	
Maternal age (months)	Maternal diet	Trial diet	*n*	Mean	sem	Mean	sem	Mean	sem	Mean	sem
2–4	CON	Chow	9	1·65	0·16	1·08	0·17	131	11	123	15
		Butter	12	2·63‡	0·14	1·75‡	0·15	162‡	12	146	19
	LP	Chow	15	1·37†	0·12	0·94	0·13	115†	10	113	17
		Butter	15	2·19‡	0·13	1·48†‡	0·13	153‡	11	138	15
6–9	CON	Chow	5	1·80	0·22	1·04	0·23	160	14	150	15
		Butter	5	2·25‡	0·22	2·02‡	0·22	189‡	12	173	14
	LP	Chow	6	1·32†	0·20	0·82†	0·21	119†	11	115	14
		Butter	7	2·15‡	0·19	1·40†‡	0·19	148†	11	159‡	13
Effect of age (*P*)				NS		NS		NS		NS	
Effect of maternal diet (*P*)				0·009		0·017		0·002		NS	
Effect of trial diet (*P*)				<0·001		<0·001		<0·001		0·012	
Interaction age × maternal diet (*P*)				NS		NS		NS		NS	
Interaction age × trial diet (*P*)				NS		NS		NS		NS	
Interaction maternal diet × trial diet (*P*)				NS		NS		NS		NS	
Interaction age × maternal diet × trial diet (*P*)				NS		NS		NS		0·031	

CON, maternal control diet, 18 % casein; LP, maternal low-protein diet, 9 % casein.

† Significant effect of maternal diet compared with animals from the same age, trial diet group (*P* < 0·05).

‡ Significant effect of trial diet compared with animals from the same maternal diet, and age group (*P* < 0·05).

**Fig. 1.**
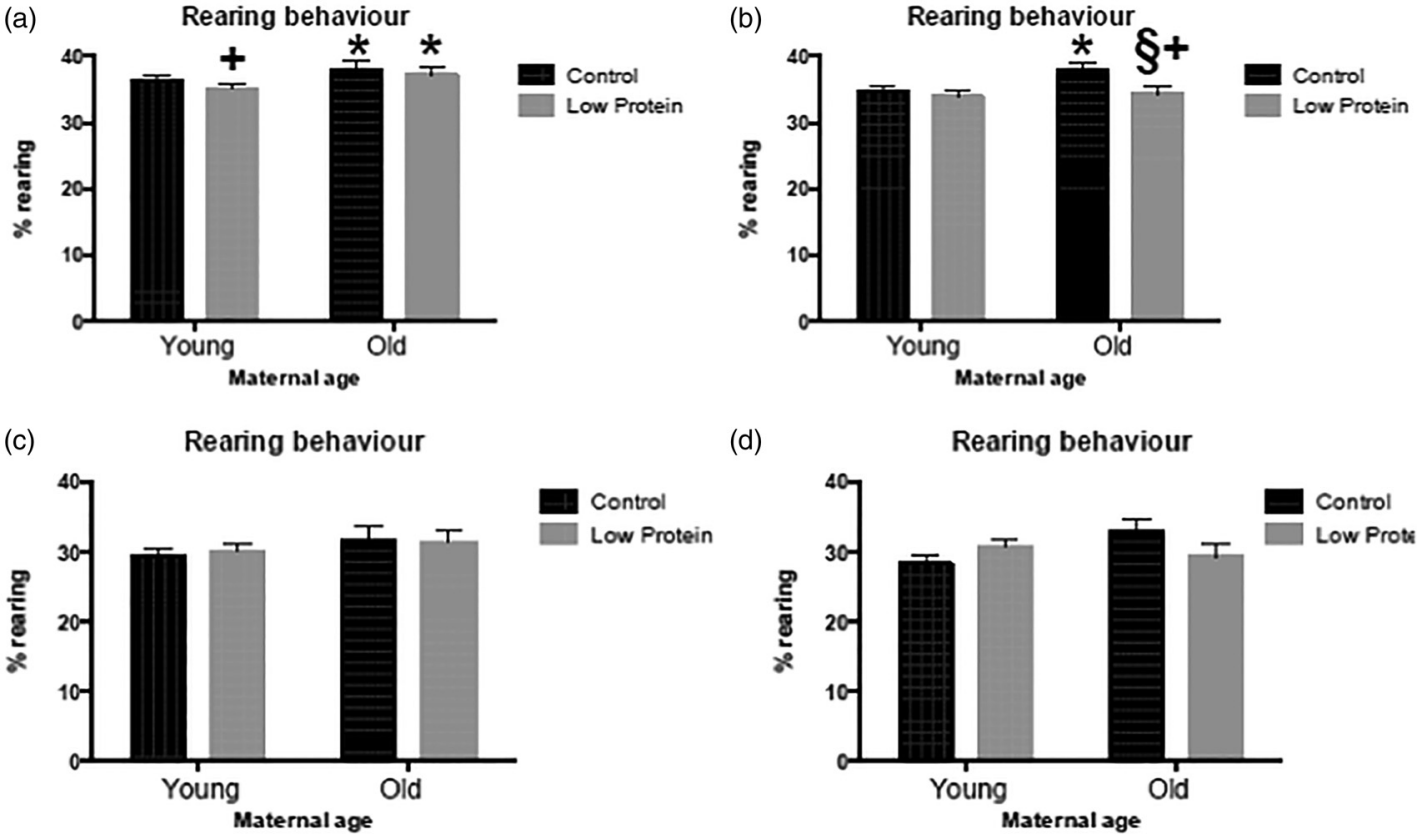
Rearing behaviour in a novel environment in: (a) chow-fed male offspring; (b) high-fat diet-fed male offspring; (c) chow-fed female offspring; (d) high-fat diet-fed female offspring. ■, Maternal control diet, 18 % casein; 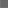, maternal low-protein diet, 9 % casein. Data are means, with their standard errors represented by vertical bars. For number of observations per group, see Tables 2 and 3. * Significant effect of maternal age compared with offspring of young mothers from the same maternal and trial diet group (*P* < 0·05). + Significant effect of maternal diet compared with animals from same maternal age and trial diet group (*P* < 0·05). § Significant effect of trial diet compared with animals from same maternal age and diet group (*P* < 0·05). Rearing in males was influenced by maternal age (*P* = 0·013), maternal diet (*P* = 0·037) and the trial diet (*P* = 0·041).

**Fig. 2.**
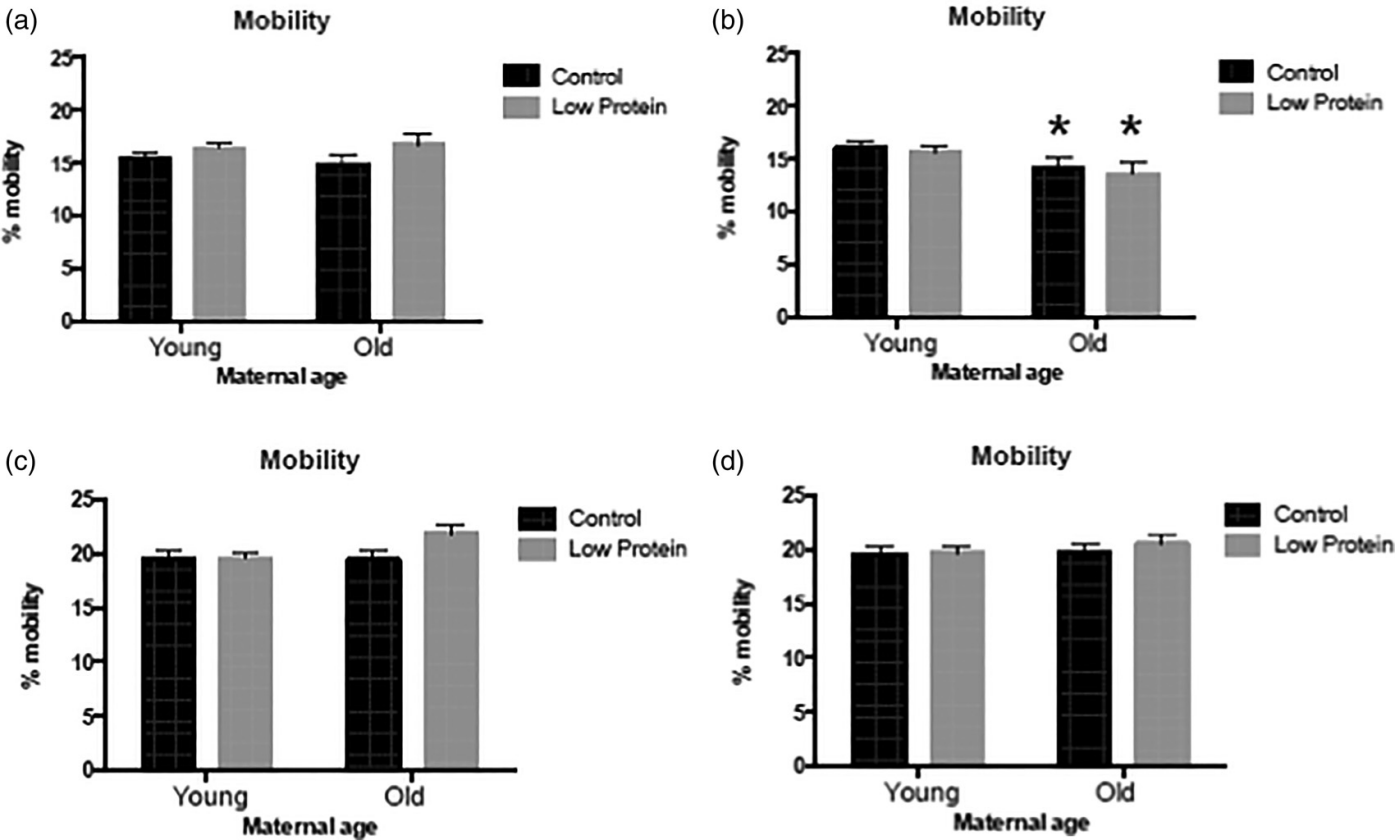
Mobility in a novel environment in: (a) chow-fed male offspring; (b) high-fat diet-fed male offspring; (c) chow-fed female offspring; (d) high-fat diet-fed female offspring. ■, Maternal control diet, 18 % casein; 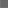, maternal low-protein diet, 9 % casein. Data are means, with their standard errors represented by vertical bars. For number of observations per group, see Tables 2 and 3. * Significant effect of maternal age compared with offspring of young mothers from the same maternal and trial diet group (*P* < 0·05). Mobility in males was influenced by maternal age (*P* = 0·007).

**Fig. 3.**
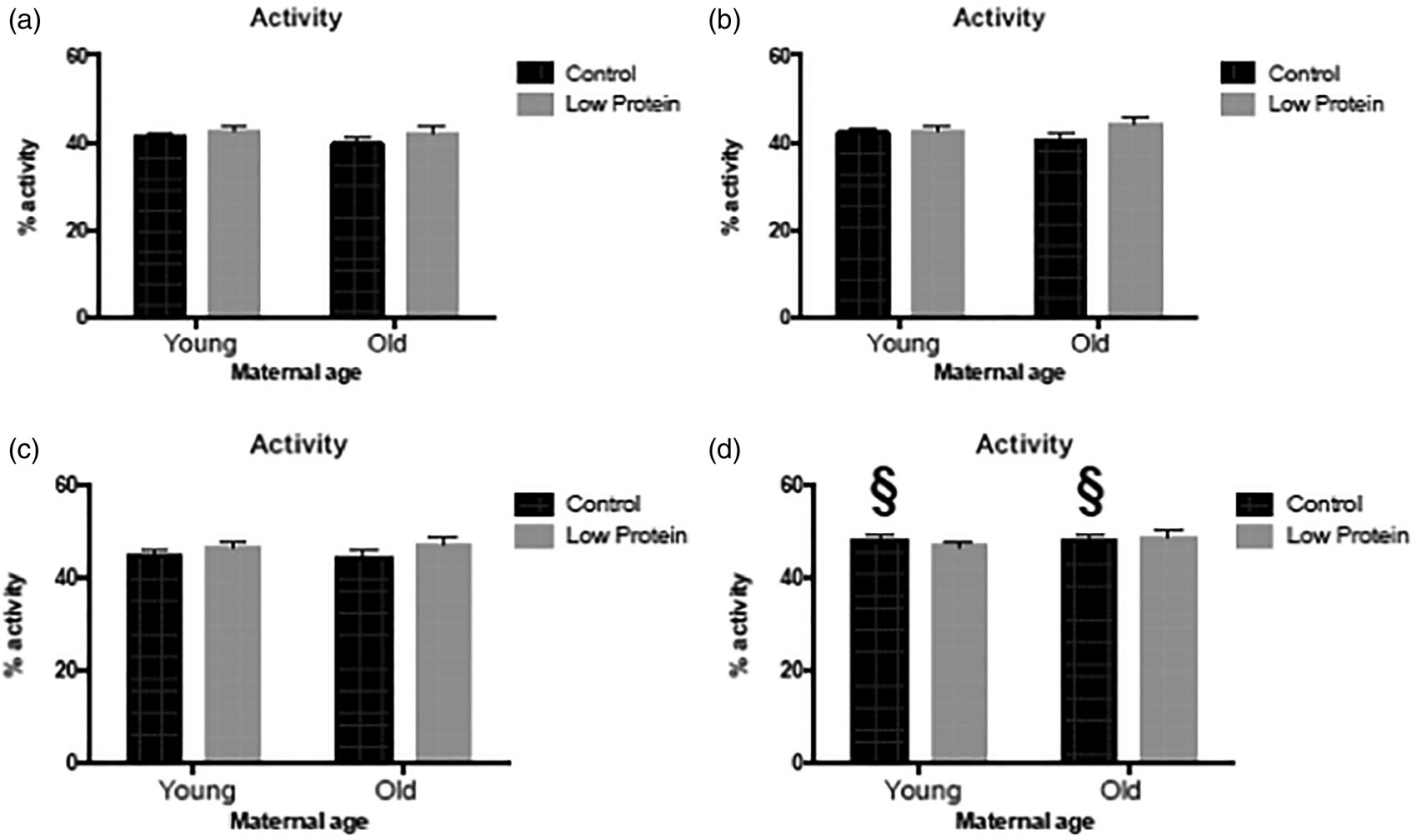
Activity in a novel environment in: (a) chow-fed male offspring; (b) high-fat diet-fed male offspring; (c) chow-fed female offspring; (d) high-fat diet-fed female offspring. ■, Maternal control diet, 18 % casein; 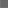, maternal low-protein. Data are means, with their standard errors represented by vertical bars. For number of observations per group, see Tables 2 and 3. § Significant effect of trial diet compared with animals from the same maternal age and diet group (*P* < 0·05). Activity in females was influenced by the trial diet (*P* = 0·041).

It was apparent from the body-weight profiles of the animals that maternal diet, maternal age and high-fat feeding all made an impact on adiposity. The resistance to fat deposition in LP-exposed animals was not fully explained by lower energy intake, so in order to determine whether differences in fat gain were explained by altered energy expenditure through physical activity, locomotor activity was determined in all animals at half way through the high-fat feeding trial. As shown in Fig. 1(c) and (d), the rearing of female offspring was not significantly influenced by maternal age, maternal diet or high-fat feeding. Similarly the mobility of females was not influenced by any of the factors under study (Fig. 2(c) and (d)), but activity was significantly increased by feeding the high-fat diet (Fig. 3(c) and (d)). Among the males, offspring of older mothers tended to rear more than those from younger mothers, whilst those exposed to the LP diet *in utero* were slightly less exploratory (Fig. 1(a)). Consumption of the high-fat diet also had a small but significant effect on rearing (Fig. 1(b)). Male offspring born to older mothers were observed to be less mobile in a novel environment (Fig. 2(a) and (b)), but this behaviour was not influenced by maternal or high-fat diet. The activity of male offspring was not influenced by maternal factors or the high-fat diet (Fig. 3(a) and (b). Thus variation in locomotor activity did not appear to explain differences in weight gain or adiposity. The difference in rearing activity was, however, an interesting behaviour for further investigation.

**Fig. 4.**
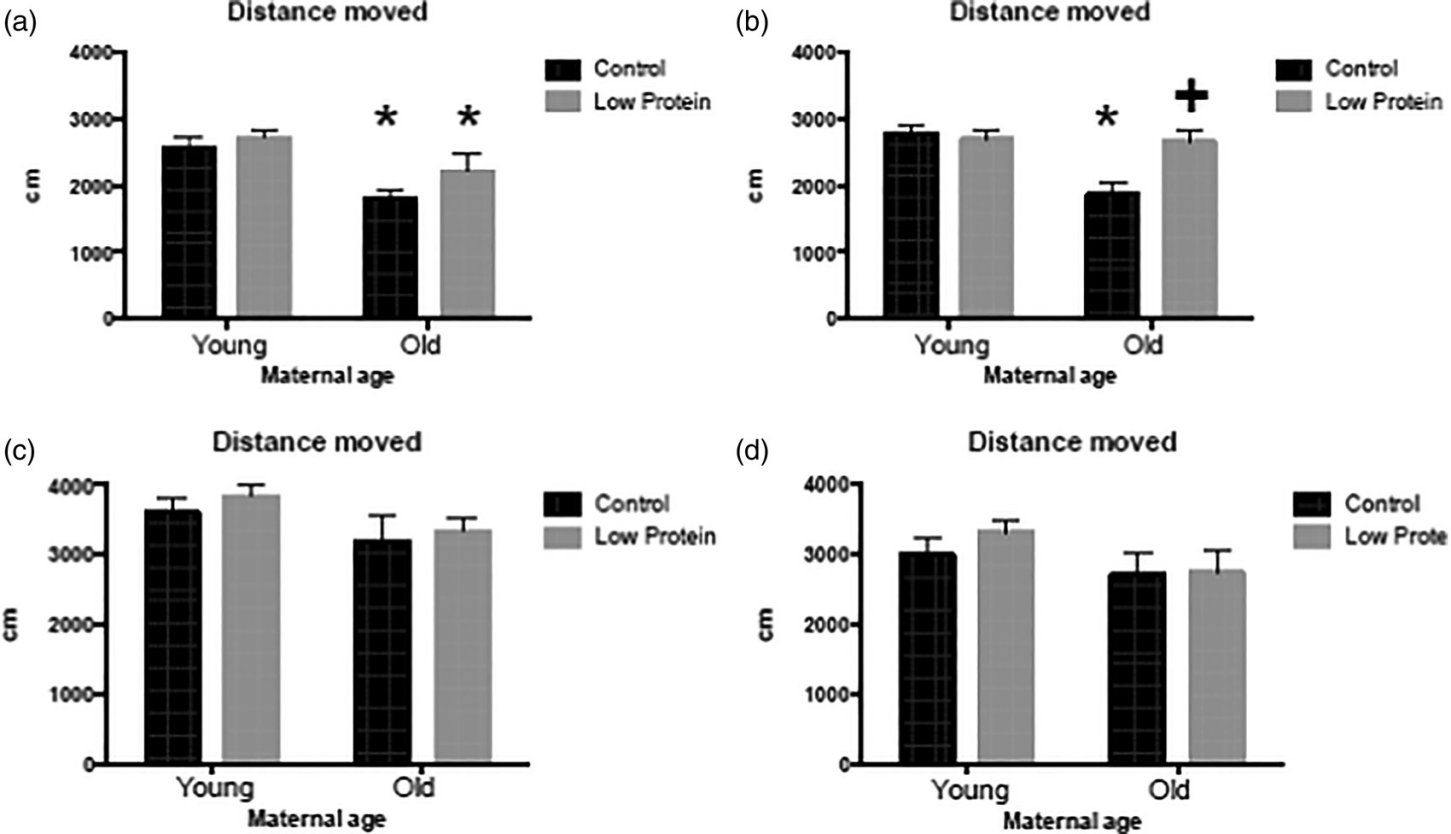
Movement on the elevated plus-maze in: (a) chow-fed male offspring; (b) high-fat diet-fed male offspring; (c) chow-fed female offspring; (d) high-fat diet-fed female offspring. ■, Maternal control diet, 18 % casein; 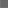, maternal low-protein. Data are means, with their standard errors represented by vertical bars. For number of observations per group, see Tables 2 and 3. * Significant effect of maternal age compared with offspring of young mothers from the same maternal and trial diet group (*P* < 0·05). + Significant effect of maternal diet compared with animals from the same maternal age and trial diet group (*P* < 0·05). In males, movement on the plus-maze was influenced by maternal diet (*P* = 0·008), maternal age (*P* < 0·001) and the interaction of maternal age and maternal diet (*P* = 0·015).

**Fig. 5.**
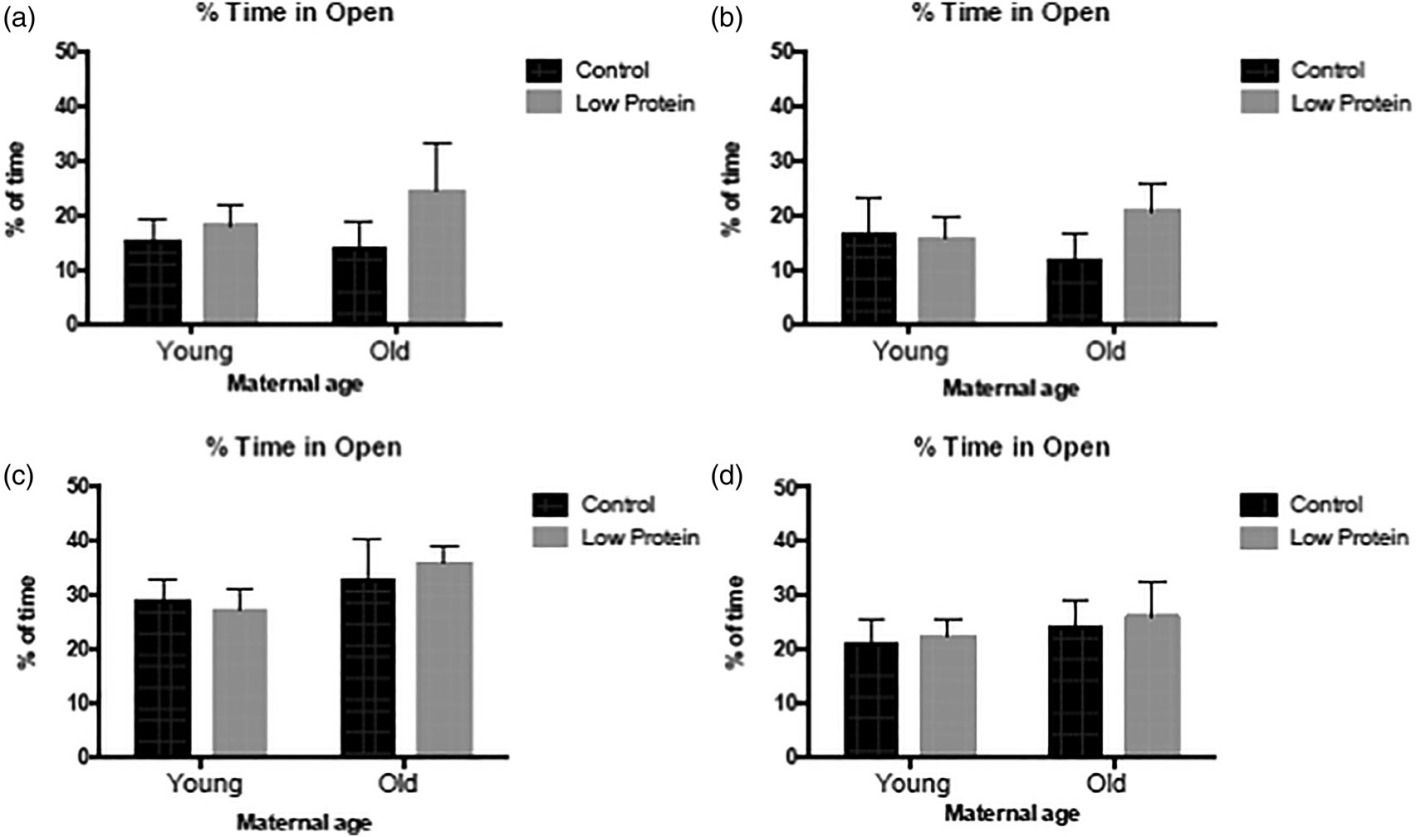
Percentage of time spent in the open arms of the elevated plus-maze in: (a) chow-fed male offspring; (b) high-fat diet-fed male offspring; (c) chow-fed female offspring; (d) high-fat diet-fed female offspring. ■, Maternal control diet, 18 % casein; 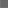, maternal low-protein. Data are means, with their standard errors represented by vertical bars. For number of observations per group, see Tables 2 and 3.

**Fig. 6.**
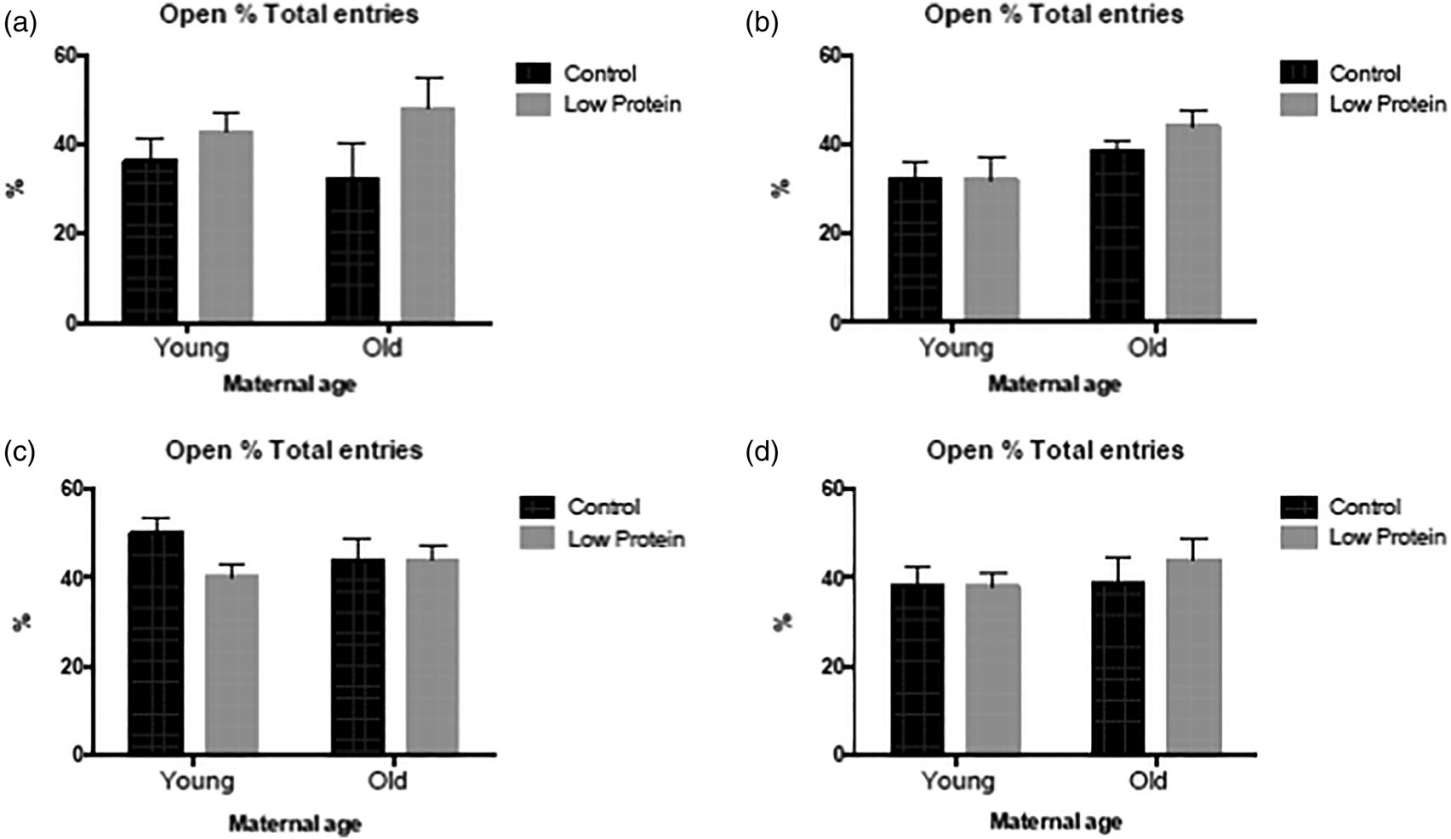
Percentage of arm entries to the open arm of the elevated plus-maze in: (a) chow-fed male offspring; (b) high-fat diet-fed male offspring; (c) chow-fed female offspring; (d) high-fat diet-fed female offspring. ■, Maternal control diet, 18 % casein; 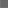, maternal low-protein. Data are means, with their standard errors represented by vertical bars. For number of observations per group, see Tables 2 and 3

Variation in rearing may be indicative of differences in exploratory behaviour. To investigate exploratory and anxiety-related behaviours animals were tested on the elevated plus-maze. The male offspring of older mothers behaved differently from the offspring of the younger dams on the elevated plus-maze. Overall the distance travelled on the maze was lower (Fig. 4(a) and (b)). These effects were partly offset by the high-fat diet (Fig. 4(b)) and maternal diet (Fig. 4(b)), as high-fat diet-fed, LP offspring from older mothers were just as active on the maze as the offspring of younger rats. The time spent in the open arms and the relative proportions of open-to-closed arm entries are measures of the exploratory and anxiety-related behaviours of the animals. There was no evidence that maternal age, diet or the high-fat diet influenced these behaviours in the male animals (Fig. 5(a) and (b); Fig. 6(a) and (b)). As shown in Figs 4–6, the feeding of the high-fat diet had no impact upon the behaviour of female offspring on the elevated plus-maze, and overall the behaviour of the females was generally unaffected by maternal age and maternal diet (with the exception of total arm entries; Fig. 5(c) and (d)). The female offspring were more active on the plus-maze than the males (Fig. 4). Rearing was scored as a behaviour on the plus-maze and among males the offspring of older mothers reared more than those from younger mothers (young 7·12 % of time rearing; old 10·24 % of time rearing; *P* < 0·001). In females the same effect of maternal age was noted but only in rats exposed to control diet *in utero* (effect of maternal age, *P* = 0·026; interaction maternal age × maternal diet, *P* = 0·042).

## Discussion

This paper reports the outcomes of a complex study design that had the capacity to explore the effects of maternal diet, maternal age and offspring diet upon body weight, adiposity and behaviour in 10-month-old rats. The work identified clear effects of maternal age in terms of reproductive success and offspring behaviour, with older mothers gaining less weight in pregnancy, having more failed pregnancies and smaller litters. Mature offspring from older mothers exhibited differences in adipocyte size and were less mobile than those from younger mothers. Maternal protein restriction resulted in lower weight gain and energy intake with high-fat feeding. The combination of greater maternal age and protein restriction was associated with the greatest resistance to weight and fat gain with a high-fat diet. Many of the effects observed were sex-specific with the impact of maternal factors being present in male but not female offspring. This was not a mechanistic study and we sought to carry out an initial, descriptive evaluation of the impact of maternal age upon the response to diet during fetal and adult life.

We have previously reported on the effects of a maternal low-protein diet upon metabolic indicators, body weight and composition in ageing rats. Bellinger *et al.*^(^^13^^)^ reported that at 9 months of age (the time at which the high-fat feeding experiment was initiated in the present study), LP-exposed males had less gonadal fat, but were of similar body weight. A significant change in phenotype occurs in protein-restricted offspring, as by 18 months of age they have larger visceral adipose tissue depots, significant lipid deposition and insulin resistance^(^^9^^)^. The present study also found smaller fat depots at 9 months, but in contrast to the previous work, the animals were also of lower body weight.

The observation that older mothers had more reproductive failure and smaller litters than the 2- to 4-month-old dams is also consistent with the literature. Older mothers commonly have smaller litters and restricted weight gain^(^^18^^)^, as noted in the present study. Little is known about the effects of greater maternal age upon the long-term physiology of rodents. In mice, offspring of older mothers were shown by Wang & vom Saal^(^^23^^)^ and Tarín *et al.*^(^^24^^)^ to be smaller than controls. Although these mouse studies found the opposite of what was observed in the present experiment, our findings were consistent with those of Ryshavskii *et al.*^(^^25^^)^ who found that offspring of older rats were heavier up to 6 weeks of age.

We primarily investigated behaviour to determine whether there were significant differences in locomotor activity that could explain the apparently differing adiposity and weight phenotypes associated with maternal undernutrition. The only differences we observed were in rearing behaviour, which has been previously reported with this model^(^^13^^)^. This behaviour may be related to anxiety and so further testing with the elevated plus-maze was undertaken. Studies of younger animals have previously shown effects of maternal age upon behaviour. Ryshavskii *et al*.^(^^25^^)^ reported that offspring of older animals were more anxious than those from younger dams when tested on the elevated plus-maze. In contrast, Zemunik *et al.*^(^^26^^)^ found that when tested in an open field, offspring of younger dams were less anxious than controls. There was little evidence that feeding the high-fat diet made an impact upon the behaviour of the animals. In rodents the feeding of a high-fat diet to induce obesity has been associated with behavioural changes that have been described as a depressive phenotype^(^^27^^)^. However, the impact of high-fat feeding is not consistent and has also been shown to reduce anxiety in male rats^(^^28^^)^ and to have varying effects that are dependent upon the materno-fetal environment during suckling^(^^29^^)^.

The present study has confirmed the resistance of rats exposed to LP *in utero* to the development of adiposity. The group of greatest interest in the present study was the male offspring of older, LP-fed mothers. These were the most resistant to weight gain with the high-fat diet and this resistance could at least in part be explained by lower energy intake and greater movement-related energy expenditure. Other factors are likely to be involved, but our previous work using indirect calorimetry has not found any impact of maternal diet upon RMR (L Bellinger and SC Langley-Evans, unpublished results; S Ware and SC Langley-Evans, unpublished results). Contrary to expectations, the feeding of the high-fat diet did not bring forward the previously identified ‘switch’ in phenotype from an obesity-resistant state to an obesity-prone insulin-resistant state, which occurs between 9 and 18 months of age^(^^9^^)^. This suggests that this is independent of diet and the metabolic state of the animal and may instead by related to programmed gene expression^(^^9^^,^^30^^)^.

Effects of maternal diet upon feeding behaviour have been reported as a common outcome of early-life programming studies in rats^(^^31^^–^^33^^)^ and this suggests that the early environment makes an impact upon the development of the hypothalamic centres that regulate food intake. Orozco-Solis *et al.* noted changes in expression of the genes in the hypothalamus, which may contribute to the regulation of food intake^(^^34^^)^. Differential expression of serotonin receptors^(^^35^^)^ may contribute to changes in feeding behaviour. Programming of brain development may also explain the observed effects of maternal diet upon locomotor behaviour^(^^36^^)^.

The data showed that the offspring of older mothers differed in several respects from those of younger mothers, with locomotor behaviour showing particularly strong effects of age. This has not been previously investigated and there are no clear explanations for the effects. However, it may be speculated that egg quality or the uterine environment for fetal development may differ between older and younger mothers. The quality of placentation or placental perfusion, for example, could significantly affect nutrient availability or endocrine signalling between mother and fetuses^(^^37^^)^. Older mothers were heavier at mating and gained less weight across their pregnancies. This may indicate that the nutrient supply to the fetuses may have been more dependent upon maternal stores than upon food intake. With more adipose tissue and muscle mass than younger mothers, these stores would be adequate to maintain fetal growth without increasing food intake, possibly at the expense of micronutrient status. The effect that this would have upon fetal development is unclear. It is also important to recognise that the offspring of the older dams represent a ‘survivor effect’, as the majority of pregnancies of the 6- and 9-month-old rats ended in failure and the lower litter sizes suggest significantly greater fetal death and resorption. Altered fetal development could be seen as adaptive responses that ensure survival, with a longer-term trade-off in terms of physiology and health^(^^38^^)^.

Maternal nutrition has long been recognised as a factor that may make an impact upon fetal development in a manner that has long-term effects on health and well-being. As contemporary trends in human nutrition and reproductive behaviour shift away from under- to overnutrition it is also important to consider the likely impact of secular trends in age of women at first birth. Across the nations of the Organisation for Economic Co-operation and Development mean age at first birth varies from 21 years (Mexico) to 30 years (UK) and has increased substantially since 1970^(^^39^^)^. In England and Wales, only 8 % of births were to women aged 35 years and over in 1990; however, in 2012, 20 % of all births were reported to be to these older women^(^^40^^)^. Mathews & Hamilton report that in the USA numbers of first births to women aged 33–39 years tripled between 1970 and 2012^(^^41^^)^. The present study is therefore timely as it suggests that independently of other factors, greater maternal age may have the capacity to programme offspring physiology, metabolism and behaviour. Much more research is required to further characterise such effects and elucidate their mechanistic basis.

The present study is limited in that it is purely descriptive of the body composition, weight and behavioural phenotypes exhibited by the animals. However, this is the first attempt to characterise the longer-term effects of maternal age upon these measures and is unique in considering the interaction of maternal dietary factors, maternal age and the adult diet in determining an age-related phenotype. It has not been possible to consider the mechanistic basis of the observations and it will now be of great interest to examine the impact of maternal diet and age upon the central control of feeding behaviour, resting energy expenditure, thermogenesis and the major metabolic pathways, including lipogenesis and lipolysis.

One of our original hypotheses was that fetal exposure to a maternal low-protein diet would result in greater weight gain and fat deposition in older rats when fed a high-fat diet. The findings of the study were not consistent with this hypothesis. Indeed, the second hypothesis that greater maternal age would exacerbate the programming effects of maternal protein restriction was also discounted, as the offspring of older, protein-restricted dams were clearly resistant to the effects of high-fat feeding. Although long-term programming of food intake and locomotor activity may partly explain this resistance to obesity the mechanisms by which maternal age and diet influence adult body composition are not fully characterised. The present study is important as it adds older maternal age to an expanding list of factors that can influence fetal development and long-term physiology and health.
